# Supplementary figures and images for: Perturbed human sub-networks by Fusobacterium nucleatum candidate virulence proteins
Source: Microbiome. 2017 Aug 10;5:89. doi: 10.1186/s40168-017-0307-1 (PMC5551000; doi:10.1186/s40168-017-0307-1)

A

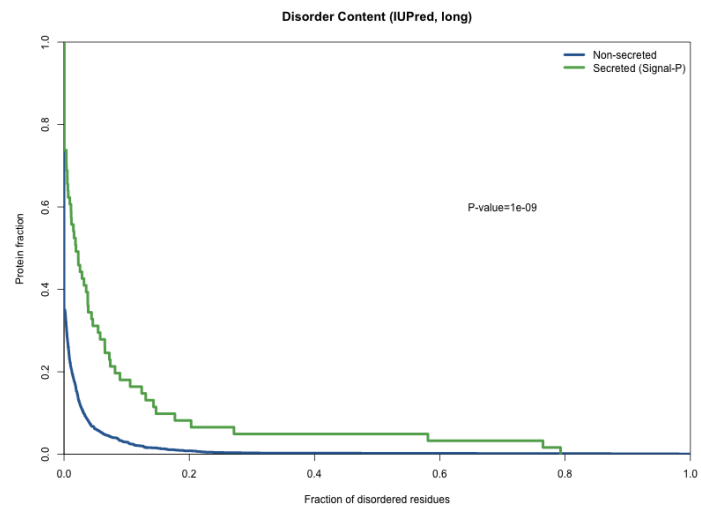

B

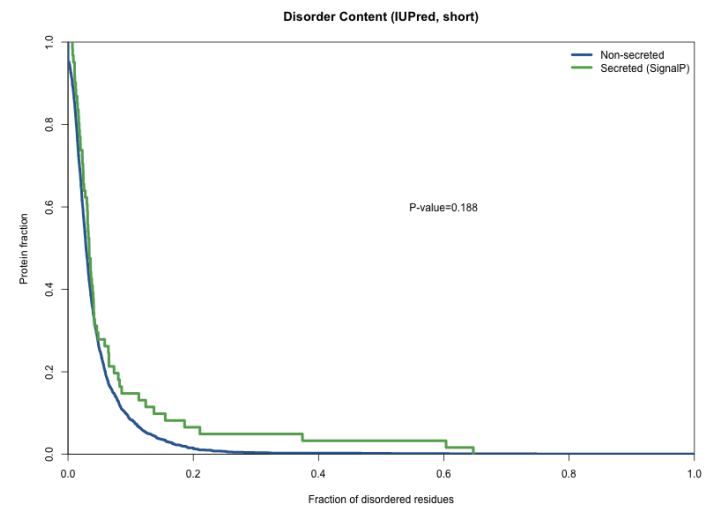

C

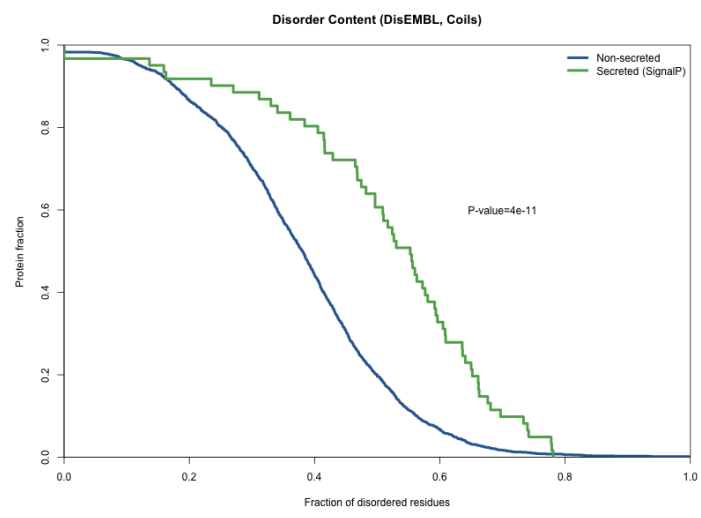

D

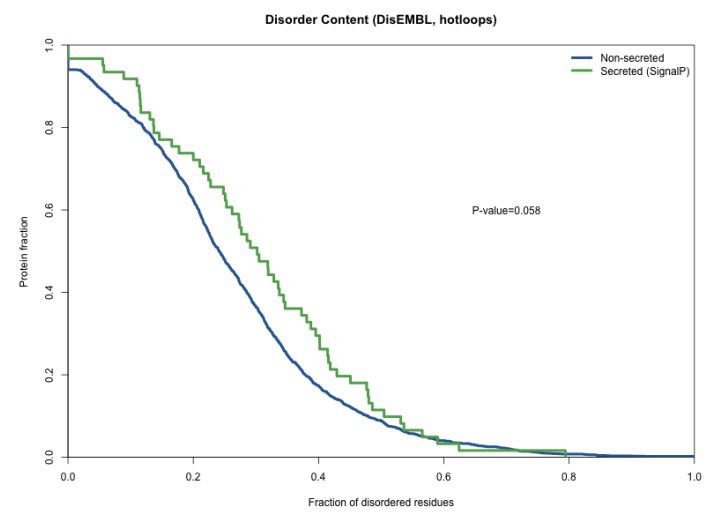

Supplement: Supplementary file 2 — Assessment of the disorder propensity of the FusoSecretome proteins (SignalP prediction) with additional prediction algorithms. (PDF 120 kb) [file 40168_2017_307_MOESM2_ESM.pdf]
