# Supplementary material for: Perturbed human sub-networks by Fusobacterium nucleatum candidate virulence proteins
Source: Microbiome. 2017 Aug 10;5:89. doi: 10.1186/s40168-017-0307-1 (PMC5551000; doi:10.1186/s40168-017-0307-1)
Supplement: Supplementary file 7 — Supplementary Information. Supplementary results, tables and figures. (PDF 1670 kb) [file 40168_2017_307_MOESM7_ESM.pdf]

Supplementary Information

Perturbed human sub-networks by *Fusobacterium nucleatum* candidate virulence proteins

Andreas Zanzoni[1], Lionel Spinelli[1], Shérazade Braham[1], Christine Brun[1,2]

[1]Aix-Marseille Université, Inserm, TAGC UMR_S1090, Marseille, France; [2]CNRS, Marseille, France.

Contents

SUPPLEMENTARY RESULTS **2**

GUT-DISEASE GENE SET ENRICHMENT USING A REDUCED STATISTICAL BACKGROUND 2

ROBUSTNESS ASSESSMENT OF THE NETWORK-BASED ANALYSIS 2

COMPARISON WITH OTHER BACTERIAL STRAINS 3

SUPPLEMENTARY TABLES **6**

SUPPLEMENTARY FIGURES **11**

REFERENCES **18**

Supplementary Results

Gut-disease gene set enrichment using a reduced statistical background

The selection of a large proteome background for the analyses on the list of inferred interactors addresses the general question "Do the interactors participate in cellular processes relevant for the microbe-host interaction?". However, the repertoire of domain-domain and SLiM-domain interaction templates is far from complete. Indeed, the total number of human proteins for which we could infer an interaction based on the templates we used (from 2013) is around 56% (11'284 proteins) of the protein-coding genes present in UniprotKB (i.e., 20'254 genes, February 2013). Therefore, we have assessed the gene set and functional enrichments using this reduced background.

The gene set enrichments still hold with the reduced background except when we consider bacterial targets alone (Table S11).

Regarding the enrichment of functional categories (BP, CC, KEGG and Reactome), a substantial fraction of enriched annotations listed in Table S6A has been identified as significantly over-represented using the reduced background as well. Namely, we found enriched: 45% of the GO Biological Processes, 53% of the GO Cellular Components, 71% of the KEGG maps and 65% of the Reactome pathways (Table S6B).

Robustness assessment of the network-based analysis

We first verified if we can obtain the same number of OCG network modules that are enriched in inferred interactors (*i.e.*, 31 modules) by shuffling proteins in the human interactome while maintaining the network structure. We performed 1'000 permutations and, as expected, we found no modules enriched in inferred interactors in any of the permuted networks (empirical P-value < 0.001).

We further assessed the robustness of interactome-based to avoid possible biases due to the presence of highly studied proteins. For this purpose, we chose an "unbiased network", built from interaction data generated by systematic yeast-two hybrid screens, hereafter named CSSB network, which we downloaded from the human interactome portal (http://interactome.dfci.harvard.edu/H_sapiens/) and that we already used in [1]) It is to note, however, that the yeast-two hybrid technique has some inherent limitations [2,3]. For instance, proteins with a strong localization signal, such as secreted proteins, seldom enter the nucleus or require specific conditions for proper folding. This leads usually to an under-representation of secreted and membrane proteins in networks built uniquely from yeast-two hybrid screens.

Since our interactome building pipeline gathered data from specialized databases (e.g., MatrixDB and InnateDB) that chiefly store interactions between extracellular and/or membrane proteins, we sought to quantify the proportion of such proteins in our interactome and in the CCSB network. To do so, we downloaded from the MatrixDB (http://matrixdb.univ-lyon1.fr/) three overlapping protein lists: (i) membrane proteins, (ii) secreted proteins and (iii) proteins annotated as components of the extracellular matrix (ECM).

Indeed, our network includes a higher proportion of ECM (2.5% compared to 1.4%) and secreted (12.2% compared to 7.5%), whereas the proportion of membrane proteins in the two networks is similar (36% compared to 31%).

Among the inferred interactors 59 are ECM proteins, 294 are annotated as secreted and 321 are associated to a membrane (union: 443 proteins, 47% of the interactors). In our interactome 41 of the ECM proteins are present (69%), but only 5 are in the CCSB network (8%). Similarly, 192 of the 294 secreted interactors are found in our interactome (65%), but only 29 are in the CCSB network (10%). Similarly, our interactome contains more interactors annotated as membrane proteins (34%) compare to the CSSB network (12%).

Nevertheless, we then repeated the network-based analyses presented in the manuscript on the CSSB network.

Only 193 inferred interactors are in the CCSB network (~21% compared to ~70% in our human interactome). Both inferred interactors average degree and betweenness are slightly higher than non-interactors but they are not significantly different (degree: 8.6 vs 6.7, P-value=0.64; betweenness: 0.0012 vs 0.0006, P-value=0.88; two-sided Mann-Whitney U test).

There is no significant enrichment of interactors participating in 2 or more network modules (i.e., multifunctional proteins). However, among the multifunctional inferred human interactors we found an enrichment of extreme multifunctional proteins (7 proteins, 5.0-fold, P-value=7.0×10^{-4}, one-sided Fisher's exact test).

We next assessed the over-representation of inferred interactors in network modules. None of them was significantly enriched. Moreover, none of the modules was enriched in gut disease genes (*i.e.*, CRC mutated, FusoExpr, CD, IBD and UC).

This discrepancy between the two analyses suggests that the higher coverage of the ECM, secreted and membrane proteins in our interactome provides a better biological context for studying the molecular interplay at the microbe-host interface. In addition, these results notably underline the methodological specificity of our approach since none of the modules in the CCSB network is found enriched in interactors, when a lack of specificity could have allowed finding some.

Comparison with other bacterial strains

We have downloaded the proteomes (UniprotKB, March 2017) and predicted the putative secretomes of 7 additional bacterial strains (see Table S12) using the SignalP and SecretomeP algorithms (Table S13). We assessed the disorder propensity of peptide-triggered secreted proteins (i.e. predicted by SignalP only) using five disorder predictors as we did for *F. nucleatum subsp nucleatum* strain (hereafter ATCC 25586). In all the additional strains, we observed a significantly higher disorder content of secreted proteins compared to non-secreted proteins with at least three predictors out of five (see Figure S2-8). This result reinforces our previous observations that secreted proteins are more disordered than non-secreted proteins.

We next compared the list of enriched Pfam domains found in the secretome of the 7 additional strains to the ATCC 22586 secretome. We indeed observed that more than a half of the ATCC 25586's enriched domains are also enriched in the other

Fusobacteria secretomes (from 54% to 82%), whereas 45% of them are found enriched in the K-12 secretome (Table S14).

Subsequently, we detected the presence of mimicry elements in the predicted secretomes that can mediate the interactions with host proteins. The vast majority of “host-like” interacting domains in ATCC 22586 strains are also found in the other *Fusobacteria* and to a lesser extent with K-12 (Table S15). Overall, we detected 150 host-like domains: 58 are present in at least two strains, 20 of them are presented in all the strains.

Regarding mimicry SLiMs, we also observed that a high fraction of ELM motifs found in ATCC 22586 are also found in other strains (from 80% to 96%, Table S15). Intriguingly, the highest fraction of ATCC 22586 mimicry motifs is found in the K-12 secretome. This can be explained by the fact the K-12 proteome is in the list of 694 proteomes of commensal and pathogenic bacteria that we used to assess the conservation of mimicry motifs found in the ATCC 22586 secretome, whereas the other *Fusobacteria* are not included. Overall, we detected 107 mimicry SLIMs, 88 of which are detected in at least two strains (48 in all 8 strains).

Starting from the list of detected mimicry domains and SLiMs, we inferred the interactions with human proteins. Sixty to sixty-six percent of the ATCC 22586 inferred interactors is found among the interactors inferred for the Fusobacteria strains and 55% are found also among K12 interactors (Table S16).

In total, we inferred the interactions with 3537 human proteins, 1884 which interact with a protein of at least two strains. Moreover, 822 human proteins are targeted by all the strains, whereas 697 are specifically targeted by proteins belonging to at least two Fusobacteria.

It is interesting to note that the number of common inferred interactors between ATCC 22586 and the other Fusobacteria strains is much higher than between ATCC 22586 and K-12, suggesting that beside a relatively small core of common interactors, these strains may have evolved specific strategies to invade and maintain themselves within the host cell. We observed a similar pattern when comparing the enrichment of functional categories (Table S17).

When mapping inferred interactors on the human interactome and assessing their over-representation in network modules, we identified 73 network modules preferentially targeted by at least one of the 8 strains. Six modules (i.e., modules 9, 74, 78, 165, 216 and 689) are preferentially targeted by all strains (Table S18). They are involved in immune response and cell-cell interactions and their constituent proteins are localized in the extracellular matrix and the cell surface. This commonality suggests that these modules and their proteins can be important players at the host-microbe interface for all the 8 strains considered. There are four modules preferentially targeted by Fusobacteria only (i.e., modules 19, 246, 577 and 615). The strain with the highest number of specifically targeted modules is K-12 with 22, followed by 22586 with 6, *vincentii 3_1_27* with 3 and animalis 4_8 with two. Interestingly, these last two show a low level of active invasion of colonic cells.

We finally checked how many of the 26 main candidate virulence proteins (i.e. network module perturbators) have potential orthologs in any of the other strains that has been classified as such by our approach. Ortholog detection is not an easy task and it goes well beyond the focus of this work. However, to identifiy putative ATCC 25586 perturbator ortholog, we first clustered the proteomes of the 8 strains at

different level of sequence identity (i.e., 90%, 50% and 40%) using the CD-HIT algorithm [4] by setting the suggested running parameters for each sequence identity threshold. We next look for the network perturbators of the other strains belonging to the same clusters of the ATCC 25586 perturbators. We found that the fraction of "conserved" pertubators is higher among Fusobacteria and varies, as expected, depending on the level of sequence identity (Table S19). Interestingly, only one ATCC 25586 perturbator is found as such among the 90 identified K-12 perturbators (Table S19).

Among "conserved" perturbators, only a small fraction shows a similar module perturbation profile. For instance, the protein coded by the ATCC 25586 gene FN0579 and its orthologs in the other Fusobacteria strains targets always the same modules (i.e. Modules 74 and 577), as well as FN1426/FN1950 and their orthologs, which target Module 246. MORN_2 containing proteins are classified as perturbators in the *vincentii* and *periodonticum* strains. The *vincentii 3_1_35A2* strain preferentially targets 4 out of 6 of the modules perturbed by the ATCC 25586 MORN_2 proteins, whereas all the others 3 modules.

Supplementary Tables

Table S11. Comparison of the enrichment of pathogen proteins targets and gut-disease related gene sets using two different statistical background.

Gene set	Full background	Reduced background
Bacterial + viral targets	1.3-fold, P-value = 1.61×10^{-11}	1.2-fold, P-value = 9.5×10^{-6}
Viral targets	1.5-fold, P-value < 2.2×10^{-16}	1.4-fold, P-value = 1.9×10^{-7}
Bacterial targets	1.1-fold, P-value = 3.5×10^{-3}	1.04-fold, P-value = 0.3
CRC secretome proteins	3.5-fold, P-value < 2.2×10^{-16}	1.6-fold, P-value = 5.7×10^{-10}
FusoExpr	2-fold, P-value=4×10^{-4}	1.7-fold, P-value = 1.2×10^{-2}
IBD	2-fold, P-value=8×10^{-4}	1.9-fold, P-value = 6.0×10^{-3}

Table S12. List of additional bacterial strains analyzed in this work.

Species and strain	Taxon ID	Body site & disease	Invasiveness in Caco-2 cells	Proteome ID	Proteome size
Fusobacterium nucleatum subsp. nucleatum 25586 [#]	190304	Oral	High	UP000002521	2046
Fusobacterium nucleatum subsp. animalis 4_8	469607	GIT, healthy biopsy	Low	UP000014361	2123
Fusobacterium nucleatum subsp. animalis 7_1	457405	Intestine inflamed, CD patient	High	UP000002799	2416
Fusobacterium nucleatum subsp. vincentii 3_1_27	469602	Rectum, healthy biopsy	Low	UP000005137	1994
Fusobacterium nucleatum subsp. vincentii 3_1_36A2	469604	Ileum inflamed, CD patient	High	UP000016231	2144
Fusobacterium periodonticum D10	620833	Oral cavity, CD patient	Not known*	UP000005809	2624
Fusobacterium periodonticum 2_1_31	469599	Colon inflamed, CD patient	Not known*	UP000003301	2371
Escherichia coli K-12	83333	-	-	UP000000625	4306

[#] Strain analyzed in the main manuscript. * The invasion potential was not tested in Caco-2 cells [5]. However, *F. periodontium* is considered as an active invader species [6].

Table S13. Size of the predicted secretomes.

Species and strain	Taxon ID	Proteome size	SignalP predicted	SecretomeP predicted	Secretome size
Fusobacterium nucleatum subsp. nucleatum 25586[#]	190304	2046	61	176	237
Fusobacterium nucleatum subsp. animalis 4_8	469607	2123	135	122	257
Fusobacterium nucleatum subsp. animalis 7_1	457405	2416	143	144	287
Fusobacterium nucleatum subsp. vincentii 3_1_27	469602	1994	142	114	256
Fusobacterium nucleatum subsp. vincentii 3_1_36A2	469604	2144	145	131	276

Fusobacterium periodonticum D10	620833	2624	172	224	396
Fusobacterium periodonticum 2_1_31	469599	2371	178	150	328
Escherichia coli K-12	83333	4306	440	353	793

[#] Strain analyzed in the main manuscript.

Table S14. Significantly enriched Pfam domains in bacterial strain secretomes.

Pfam entry	nucleatum atcc 25586	animalis 7_1	animalis 4_8	vincentii 3_1_36A2	vincentii 3_1_27	periodonticum D10	periodonticum 2_1_31	E. coli K-12
Autotransporter	x	x	x	x	x	x	x	x
DUF3798		x						
DUF4198		x	x	x	x			
FMN_bind		x	x	x	x			
FadA						x	x	
Fil_haemagg_2	x		x	x	x	x	x	
MORN_2	x	x		x	x	x	x	
OEP		x	x			x		x
OmpA			x	x	x		x	
PepSY				x		x	x	
Peripla_BP_2						x		x
POR_N	x							
Plug	x	x	x	x	x	x		x
SBP_bac_3		x	x					x
SBP_bac_5	x	x	x	x	x	x	x	x
SBP_bac_7				x				
SH3_3		x	x					
ShlB	x			x	x			
Surf_Ag_VNR	x	x	x	x	x	x	x	x
TonB_dep_Rec	x	x		x	x	x		x
TPR_11	x							
YadA_anchor		x	x	x	x			
YadA_head		x						
YadA_stalk	x	x	x	x	x	x	x	

x = enriched (one-sided Fisher's test, corrected P-value<0.1)

Table S15. Percentages of *F. nucleatum subps. nucleatum* (ATCC 22586) host-like domains and mimicry motifs found in the other bacterial strains.

**Strain**	**Percentage of atcc 22586 "host-like" domains found in other strains**	**Percentage of atcc 22586 mimicry SLiMs found in other strains**
F.nucleatum subsp. animalis 7_1	81%	88%
F.nucleatum subsp. animalis 4_8	79%	84%
F.nucleatum subsp. vincentii 3_1_36A2	89%	86%
F. nucleatum subsp. vincentii 3_1_27	89%	80%
F. periodonticum D10	77%	80%
F.periodonticum 2_1_31	77%	88%
E. coli K-12	52%	96%

Table S16. Percentages of *F. nucleatum subps. nucleatum* (ATCC 22586) inferred interactors found in the other bacterial strains.

Strain	Inferred interactors (interactions)	Percentage of atcc 22586 inferred interactors found for other strains
F.nucleatum subsp. animalis 7_1	1711 (6322)	60%
F.nucleatum subsp. animalis 4_8	1559 (6038)	60%
F.nucleatum subsp. vincentii 3_1_36A2	1460 (5897)	63%
F. nucleatum subsp. vincentii 3_1_27	1446 (5603)	66%
F. periodonticum D10	1654 (6657)	61%
F.periodonticum 2_1_31	1586 (6469)	62%
E. coli K-12	2764 (19'986)	55%

Table S17. Percentages of *F. nucleatum subps. nucleatum* (ATCC 22586) enriched functional categories found in the other bacterial strains.

Strain	Percentage of atcc 22586 enriched annotations found in other strains			
	BP	*CC*	*KEGG*	*Reactome*
F.nucleatum subsp. animalis 7_1	58%	60%	51%	58%
F.nucleatum subsp. animalis 4_8	58%	53%	53%	57%
F.nucleatum subsp. vincentii 3_1_36A2	61%	67%	64%	56%
F. nucleatum subsp. vincentii 3_1_27	61%	53%	62%	56%
F. periodonticum D10	64%	57%	62%	56%
F.periodonticum 2_1_31	71%	53%	67%	61%
E. coli K-12	66%	63%	78%	57%

Table S18. List of preferentially targeted modules by candidate virulence proteins in all the bacterial strains.

Module	nucleatum atcc 25586	animalis 7_1	animalis 4_8	vincentii 3_1_36A2	vincentii 3_1_27	periodonticum D10	periodonticum 2_1_31	E. coli K-12
4								x
9	x	x	x	x	x	x	x	x
16	x	x	x			x	x	
18		x	x			x	x	
19	x	x	x	x	x	x	x	
25		x	x			x	x	
33								x
37		x				x		
38		x	x			x	x	
40		x	x			x	x	
42	x	x		x	x	x	x	x
44				x	x	x	x	
58		x		x	x	x	x	
65								x
74	x	x	x	x	x	x	x	x

78	x	x	x	x	x	x	x	x
83								x
85								x
87								x
89	x			x	x			
90	x							
106		x		x	x	x	x	
110								x
120		x	x	x	x	x	x	x
138	x	x	x				x	
158								x
165	x	x	x	x	x	x	x	x
191								x
194	x			x			x	x
216	x	x	x	x	x	x	x	x
218		x		x	x	x	x	
225					x			
246	x	x	x	x	x	x	x	
277	x							
290								x
293		x		x	x	x	x	x
298	x							
300	x			x	x		x	x
367					x			
371	x				x			
386		x	x	x	x	x	x	x
389								x
392			x				x	
400					x			
405								x
433	x							x
447		x		x	x	x	x	
451	x							
455			x		x			
456	x	x		x	x	x	x	
480								x
491								x
493		x		x	x		x	x
563	x			x	x		x	
565					x			x
571	x		x	x	x	x	x	
577	x	x	x	x	x	x	x	
581								x
587	x				x			
615	x	x	x	x	x	x	x	
625	x						x	
658				x	x		x	
672		x	x	x	x	x	x	x
688				x	x			
689	x	x	x	x	x	x	x	x
702	x	x	x		x	x	x	
722								x
739								x
745	x							
752								x
757			x					
771								x
772								x
783						x	x	
785				x	x		x	x
794	x			x	x			
804			x					
831	x							
834								x

x = enriched (one-sided Fisher’s test, corrected P-value < 0.025)

Table S19. Number of “conserved” *F. nucleatum subps. nucleatum* (ATCC 22586) perturbators in the other bacterial strains.

Strain	Network perturbators (Z-score>2)	Number (%) of “conserved” atcc 22586 perturbators in other strains		
		90% identity	*50% identity*	*40% identity*
F.nucleatum subsp. animalis 7_1	15	3 (11%)	6 (23%)	7 (27%)
F.nucleatum subsp. animalis 4_8	17	5 (19%)	6 (23%)	7 (27%)
F.nucleatum subsp. vincentii 3_1_36A2	27	7 (27%)	9 (35%)	10 (38%)
F. nucleatum subsp. vincentii 3_1_27	26	6 (23%)	9 (35%)	9 (35%)
F. periodonticum D10	23	2 (8%)	8 (31%)	9 (35%)
F.periodonticum 2_1_31	27	2 (8%)	6 (23%)	8 (31%)
E. coli K-12	90	0	1 (4%)	1(4%)

Supplementary Figures

Figure S2. Assessment of the disorder propensity of the *F. nucleatum subsp. animalis 4_8* secreted proteins (SignalP prediction).

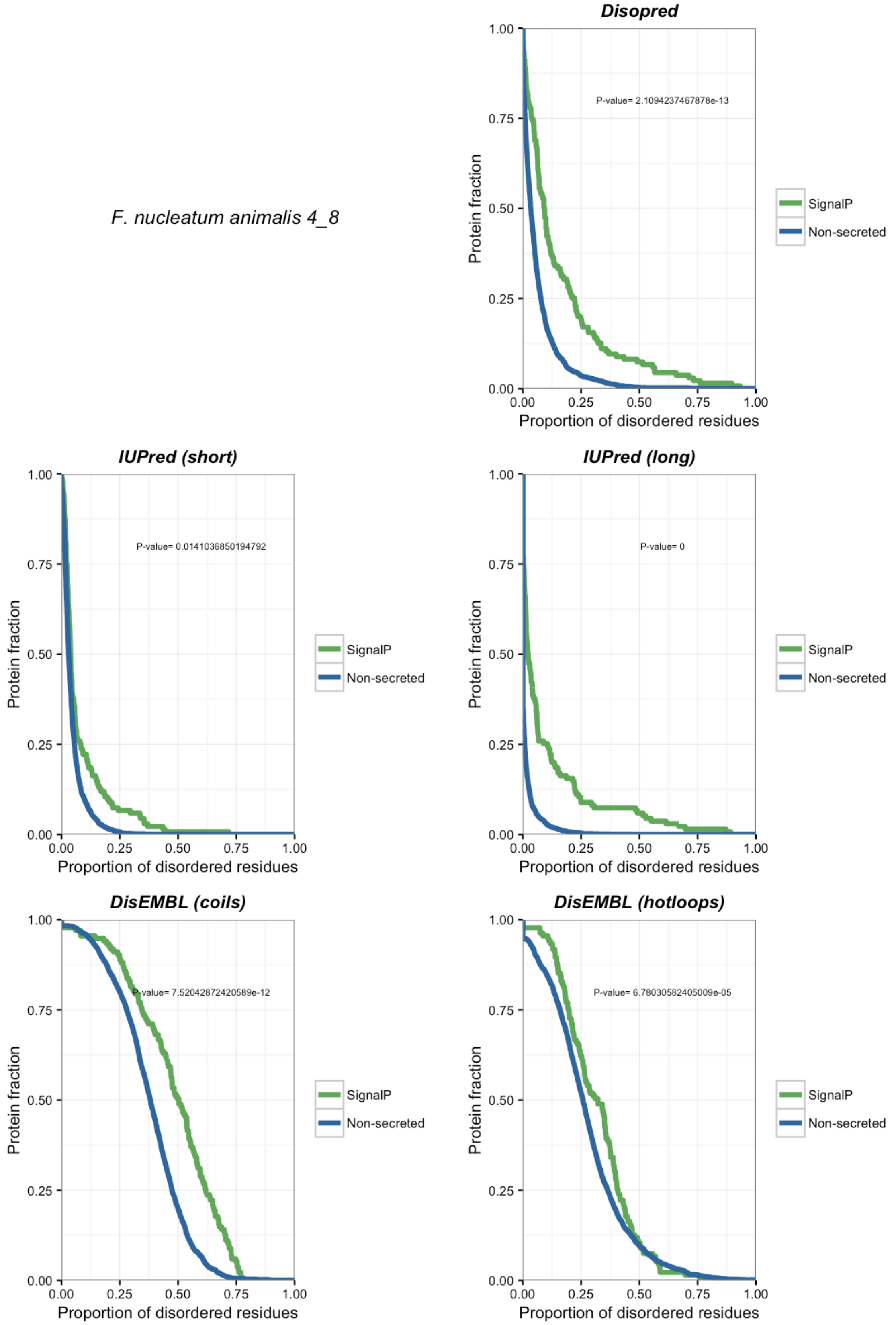

Figure S3. Assessment of the disorder propensity of the *F. nucleatum subsp. animalis 7_1* secreted proteins (SignalP prediction).

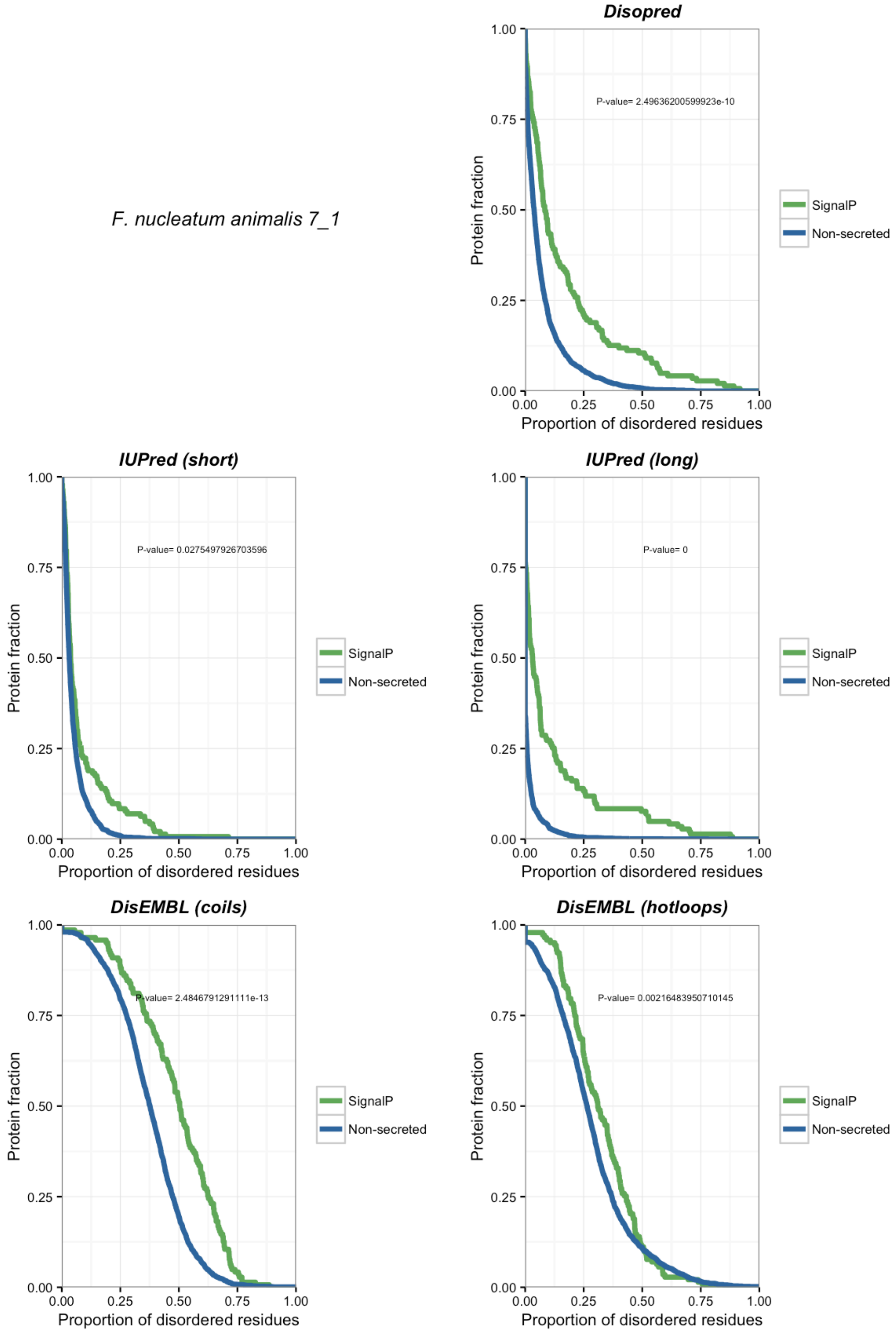

Figure S4. Assessment of the disorder propensity of the *F. nucleatum subsp. vincentii 3_1_27* secreted proteins (SignalP prediction).

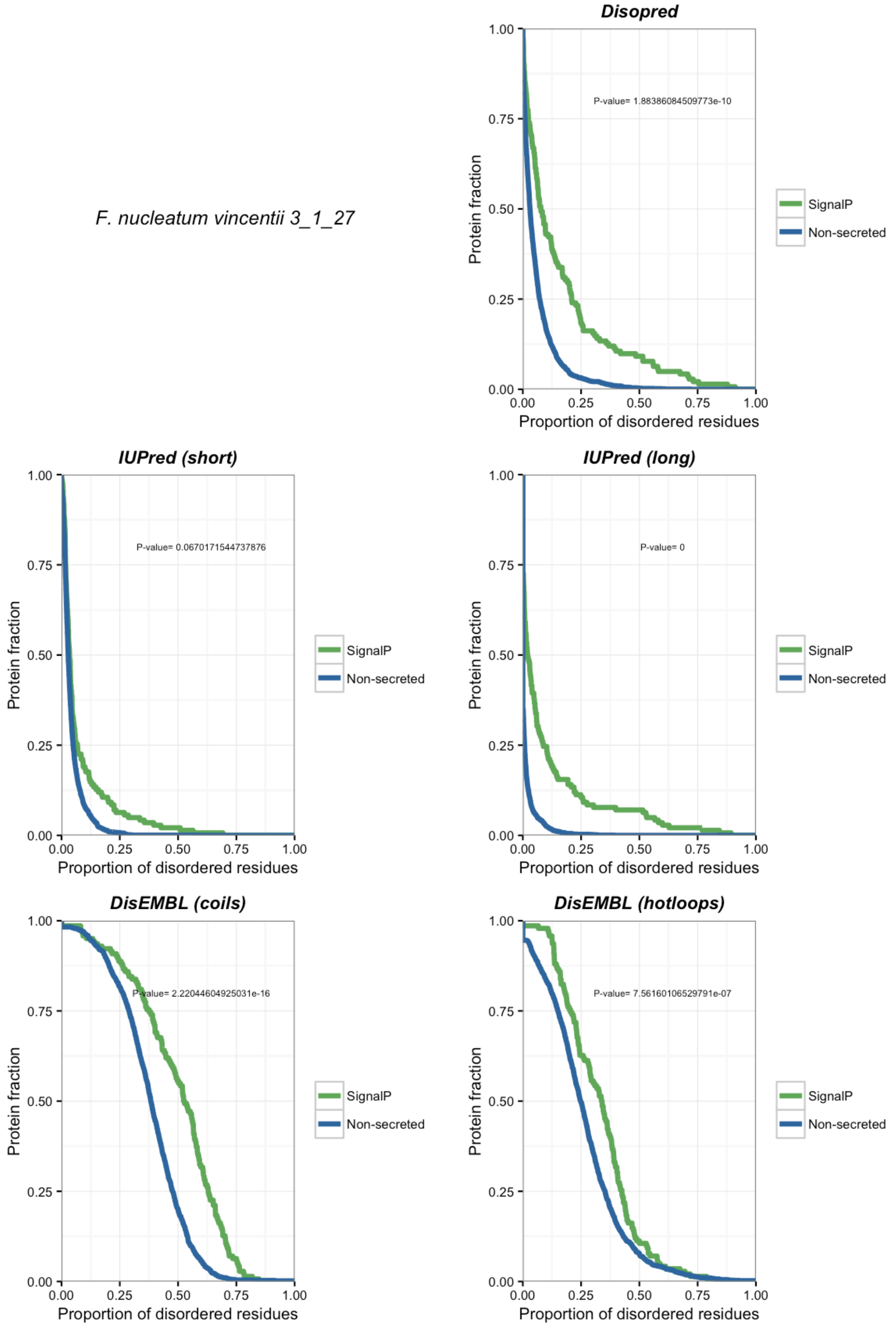

Figure S5. Assessment of the disorder propensity of the *F. nucleatum subsp. vincentii 3_1_36A* secreted proteins (SignalP prediction).

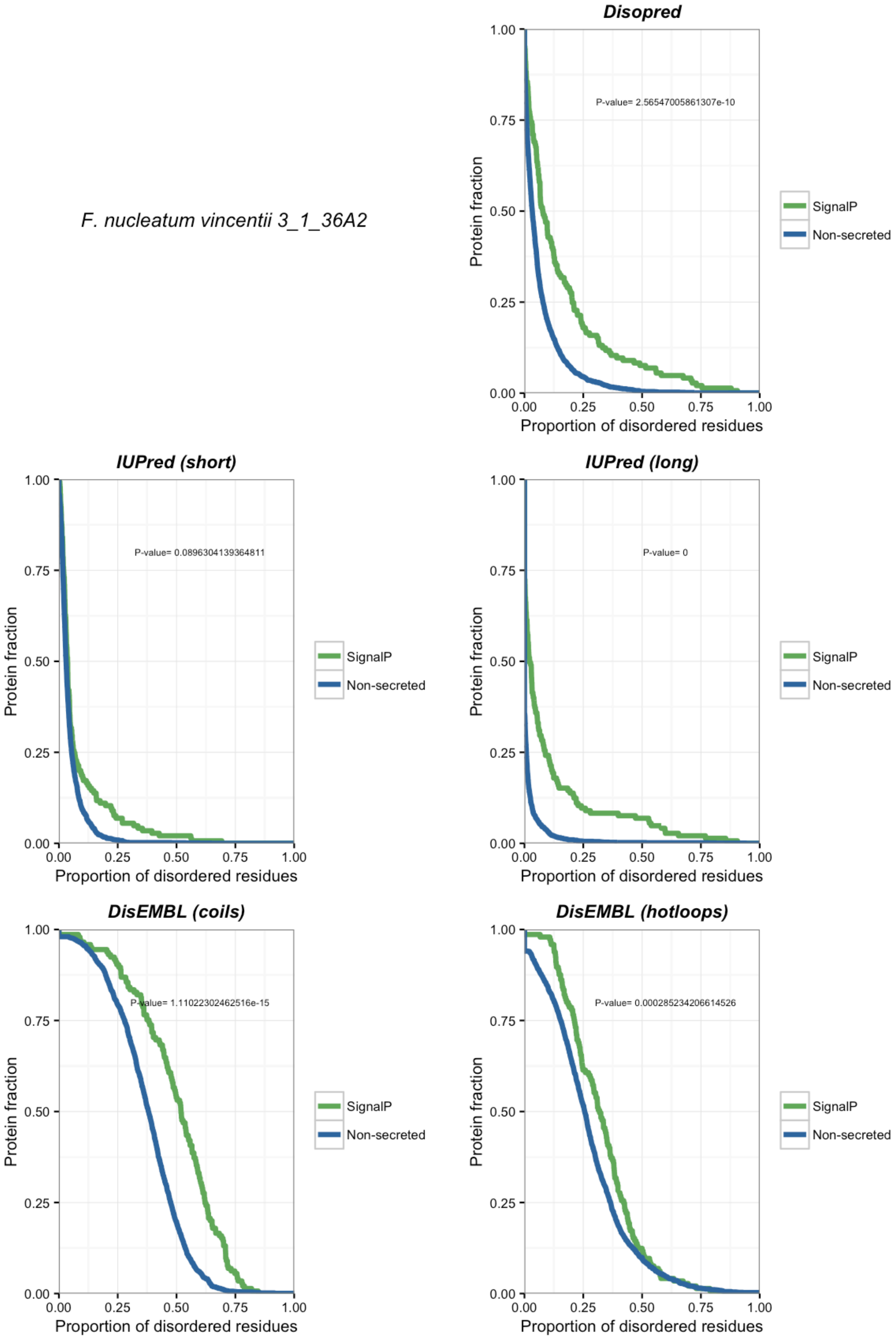

Figure S6. Assessment of the disorder propensity of the *F. periodonticum D10* secreted proteins (SignalP prediction).

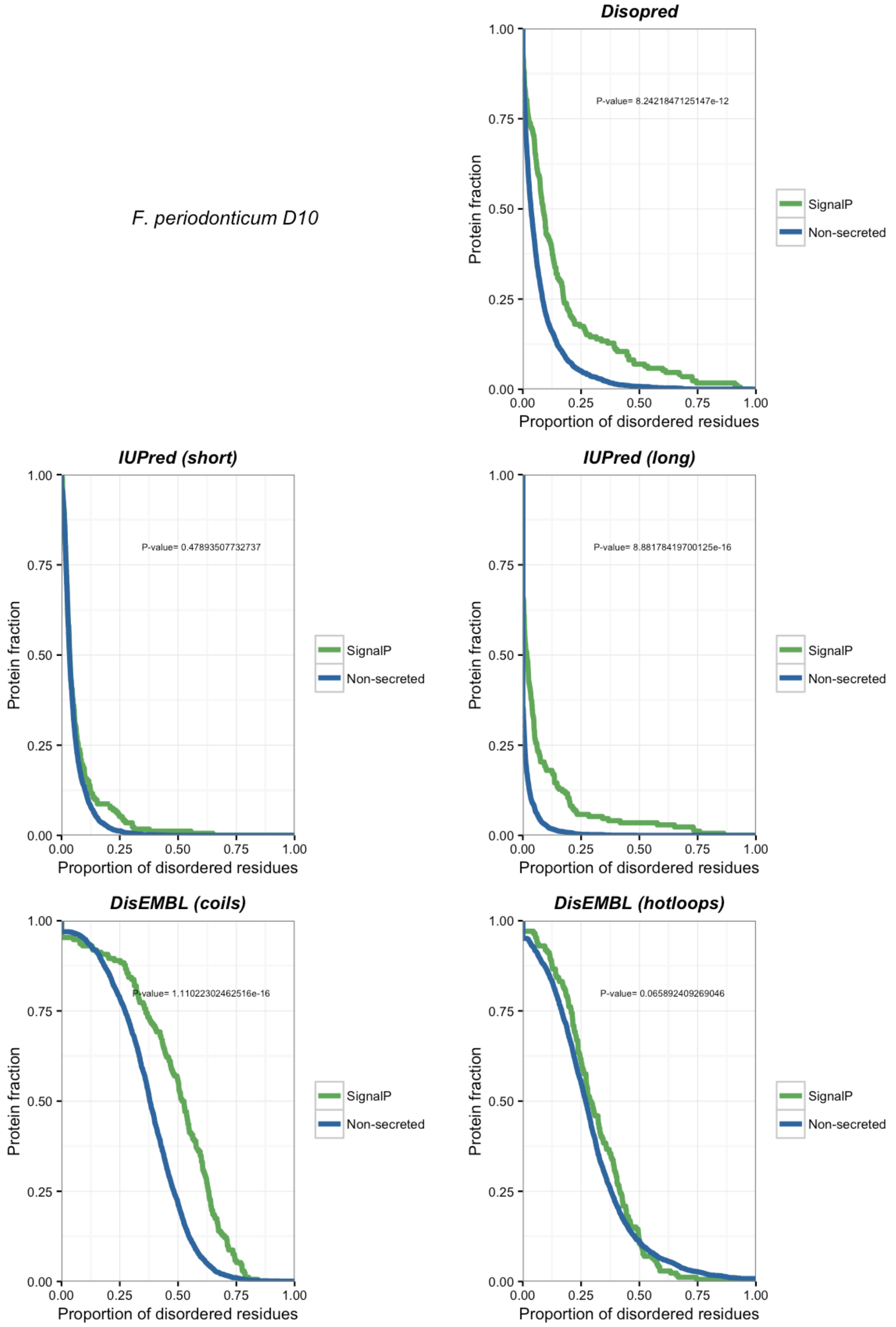

Figure S7. Assessment of the disorder propensity of the *F. periodonticum 2_1_31* secreted proteins (SignalP prediction).

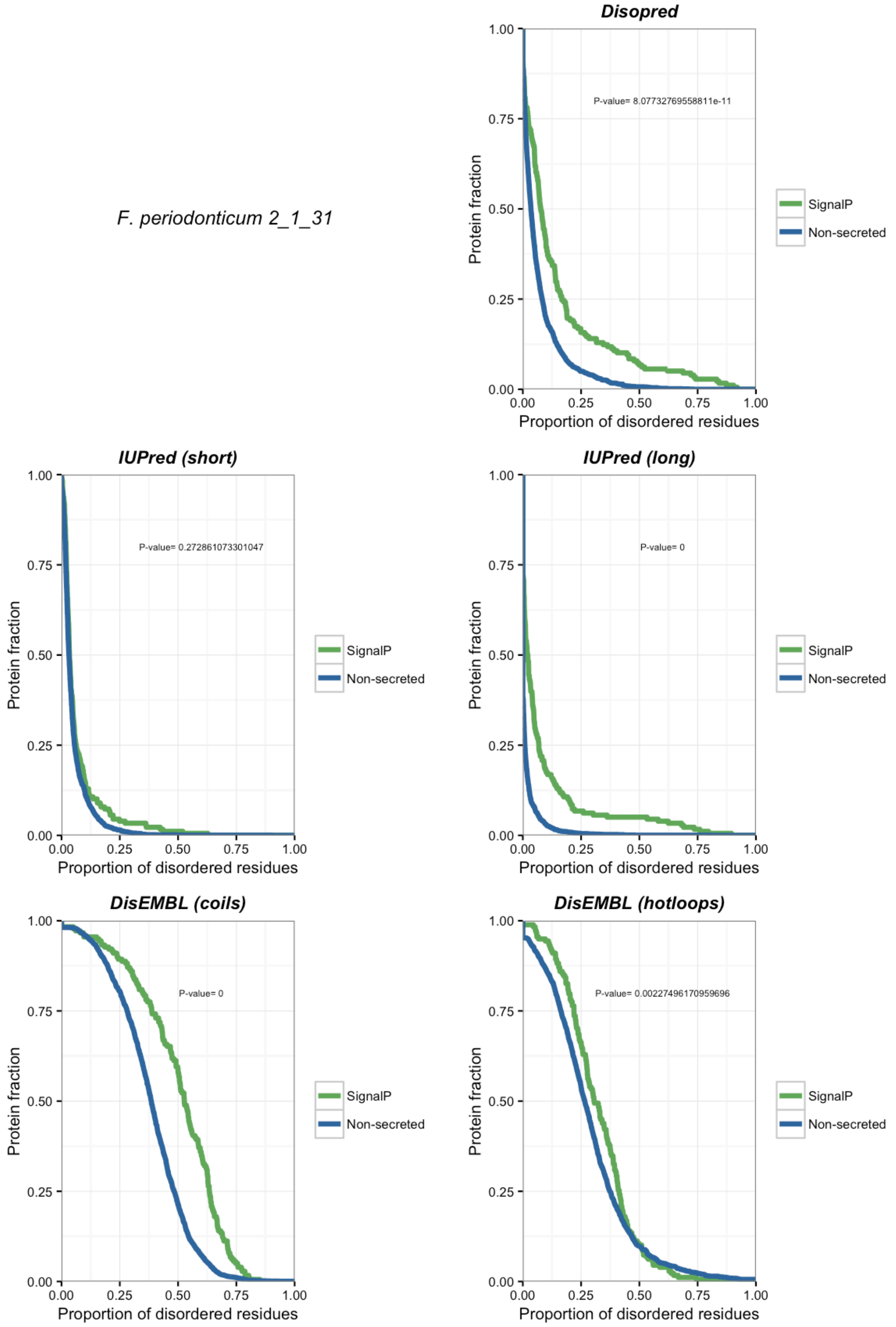

Figure S8. Assessment of the disorder propensity of the *E. coli K-12* secreted proteins (SignalP prediction).

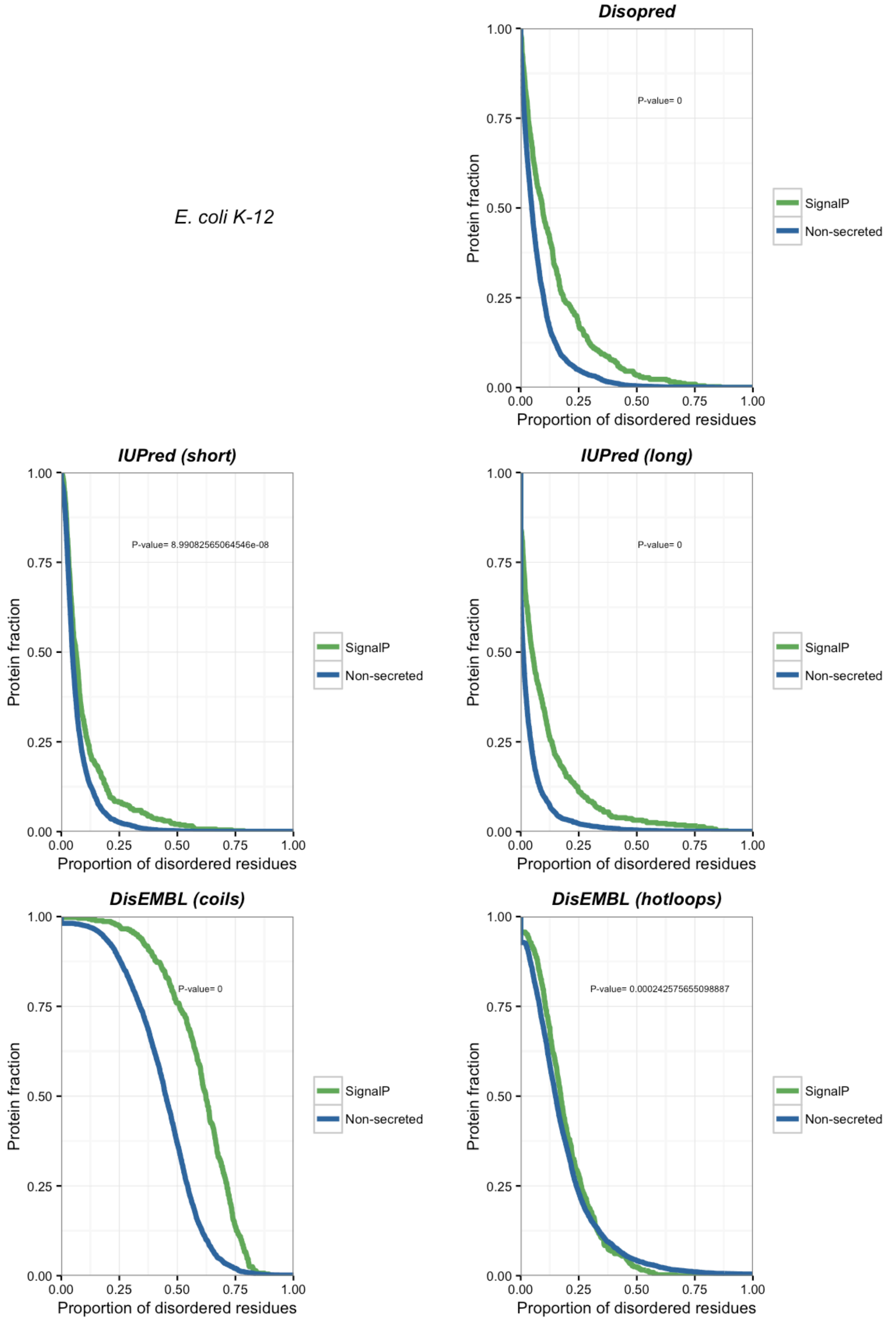

References

1. Chapple CE, Robisson B, Spinelli L, Guien C, Becker E, Brun C. Extreme multifunctional proteins identified from a human protein interaction network. Nat. Commun. 2015;6:7412.

2. Koegl M, Uetz P. Improving yeast two-hybrid screening systems. Brief. Funct. Genomic. Proteomic. 2007;6:302–12.

3. Stynen B, Tournu H, Tavernier J, Van Dijck P. Diversity in genetic in vivo methods for protein-protein interaction studies: from the yeast two-hybrid system to the mammalian split-luciferase system. Microbiol. Mol. Biol. Rev. MMBR. 2012;76:331–82.

4. Fu L, Niu B, Zhu Z, Wu S, Li W. CD-HIT: accelerated for clustering the next-generation sequencing data. Bioinforma. Oxf. Engl. 2012;28:3150–2.

5. Strauss J, Kaplan GG, Beck PL, Rioux K, Panaccione R, Devinney R, et al. Invasive potential of gut mucosa-derived Fusobacterium nucleatum positively correlates with IBD status of the host. Inflamm. Bowel Dis. 2011;17:1971–8.

6. Manson McGuire A, Cochrane K, Griggs AD, Haas BJ, Abeel T, Zeng Q, et al. Evolution of invasion in a diverse set of Fusobacterium species. mBio. 2014;5:e01864.
